# Single-Incision Combined Laparoscopic Right Hemicolectomy and Cholecystectomy: A Case Report

**DOI:** 10.7759/cureus.71083

**Published:** 2024-10-08

**Authors:** Ayub Ansari, Stephanie Yoo, Ali Thahab, Feng Ming Li, Huy T Nguyen

**Affiliations:** 1 Surgery, Kansas City University of Medicine and Biosciences, Kansas City, USA; 2 Surgery, Advanced Surgical Associates, Santa Clara, USA; 3 Medicine, Kansas City University of Medicine and Biosciences, Kansas City, USA

**Keywords:** cholecystectomy laparoscopic, laparoscopic colorectal surgery, right-sided hemicolectomy, single-incision laparoscopic surgery, surgical case reports

## Abstract

Minimally invasive surgery has transformed the management of complex procedures, offering reduced postoperative pain, faster recovery time, and improved cosmetic outcomes. Despite the growing use of minimally invasive techniques, literature specifically addressing single-incision laparoscopic surgery (SILS) for combined hemicolectomy and cholecystectomy is limited. This report seeks to fill this gap by detailing the successful management of a patient case through a single-incision combined laparoscopic right hemicolectomy and cholecystectomy. A 66-year-old female was referred to surgical consultation following a routine screening colonoscopy that identified a greater than 5 cm sessile polyp in the ascending colon. A follow-up computed tomography (CT) scan of the abdomen and pelvis revealed a non-metastatic mass in the ascending colon and gallstones correlating with the patient's reported abdominal discomfort. The decision was made to proceed with a single-incision laparoscopic right hemicolectomy and cholecystectomy. The cholecystectomy and subsequent right hemicolectomy were both performed through a 3 cm umbilical incision using the advanced access platform. Postoperative recovery was uneventful, with the patient passing flatus by day three, starting a clear liquid diet, and being discharged by day four. Pathological analysis of specimens revealed chronic cholecystitis with cholelithiasis and a tubulovillous adenoma of the colon without high-grade dysplasia or metastatic carcinoma. At the 15-day follow-up, the patient reported a full resumption of normal activities and was highly satisfied with the cosmetic results. This case report highlights the benefits of combining SILS right hemicolectomy and cholecystectomy through reducing multiple abdominal procedures, surgical trauma, operating time, and recovery period, all while achieving excellent cosmetic outcomes. Further research and advanced training in SILS combined procedures are needed for broader applicability in more complex cases.

## Introduction

Minimally invasive surgery has transformed the management of complex procedures, offering reduced postoperative pain and improved cosmetic outcomes while maintaining standard operating times. Single-incision laparoscopic surgery (SILS) is an advancement in this field, especially in colorectal surgery, where it allows procedures such as hemicolectomy, cholecystectomy, appendectomy, and combined procedures to be performed through a single umbilical incision [1]. This approach reduces the number and size of surgical incisions, recovery time, and visits to the operating room in the case of combined procedures [2]. While traditional laparoscopic procedures are effective, they involve multiple incisions, multiplying the risks of wound complications, incisional hernias, and unsatisfactory cosmetic outcomes [3]. SILS mitigates these risks but presents additional challenges, such as loss of triangulation and increased technical difficulties that require advanced surgical skills [4].

This case report details the successful management of a patient case through a single-incision combined laparoscopic right hemicolectomy and cholecystectomy. The rarity of combined SILSs in such contexts, combined with the detailed description of the technique, underscores the significance of this report. Despite the growing use of minimally invasive techniques, literature specifically addressing SILS for combined hemicolectomy and cholecystectomy is limited [5]. This report aims to address this gap by contributing to the expanding evidence on the effectiveness of SILS in managing patients with multiple abdominal conditions and significant comorbidities.

## Case presentation

A 66-year-old female was referred to surgical consultation following a routine screening colonoscopy that identified a greater than 5 cm sessile polyp in the ascending colon. Due to the size of the polyp and the associated risk of perforation, complete excision was not achieved during the colonoscopy. The polyp site was marked with India ink proximally and distally for future surgical reference, and further histopathological analysis of the fragmented polypectomy specimens revealed tubulovillous adenoma.

The patient's medical history is significant for hypertension and hyperlipidemia, with no previous surgical history and non-contributory family history. She is a nonsmoker and denies any alcohol use. The patient reported intermittent right-sided abdominal discomfort for the past several months but did not prompt an earlier investigation. The physical examination revealed a well-oriented and alert patient with stable vital signs. The cardiovascular and respiratory systems were unremarkable, and the abdominal examination showed a soft, non-tender abdomen with normoactive bowel sounds.

**Figure 1:**
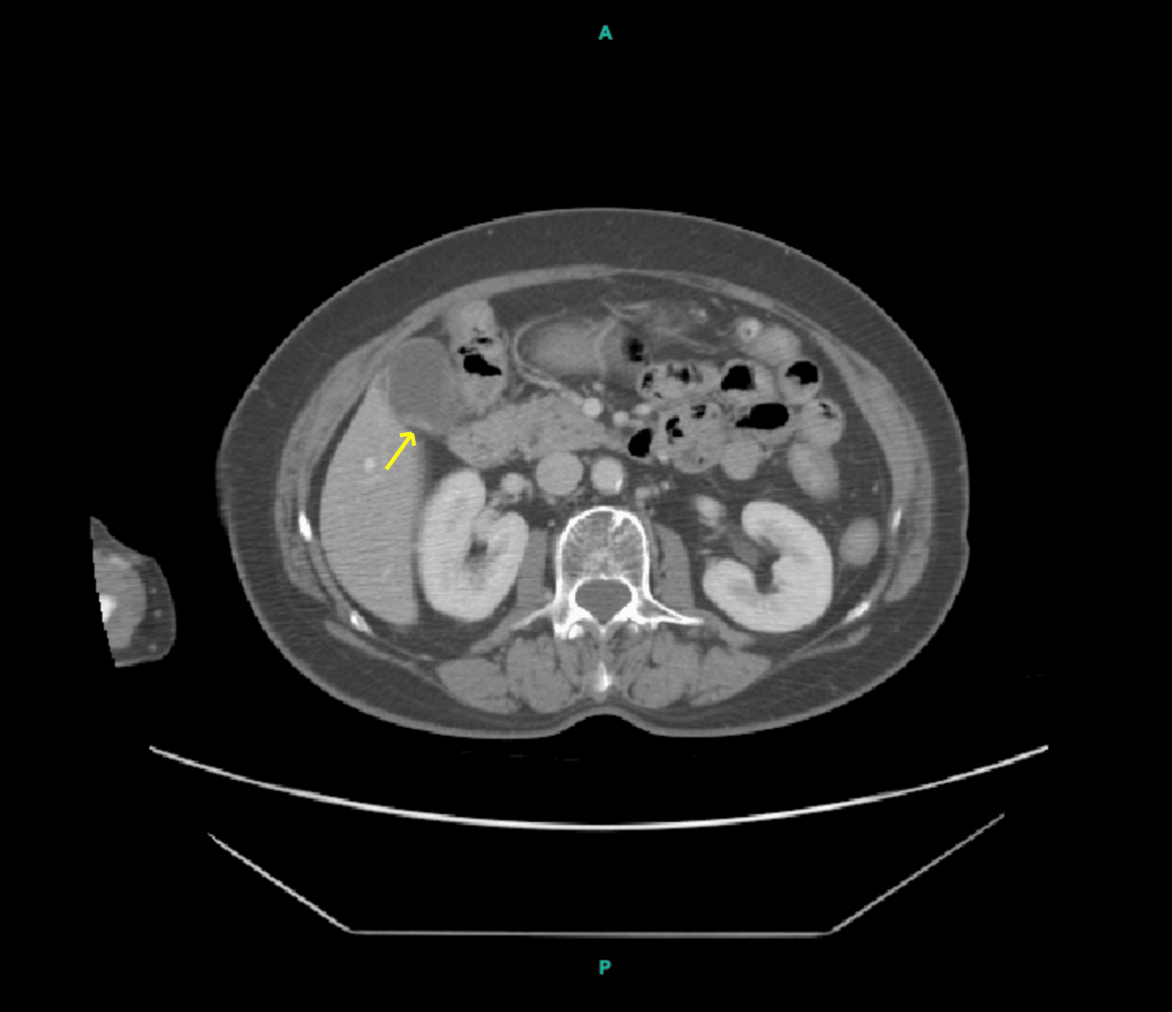
Axial CT scan of the abdomen demonstrating the gallbladder with multiple gallstones. The yellow arrow points to clustered hyperdense calcifications within the gallbladder lumen, consistent with gallstones.

**Figure 2:**
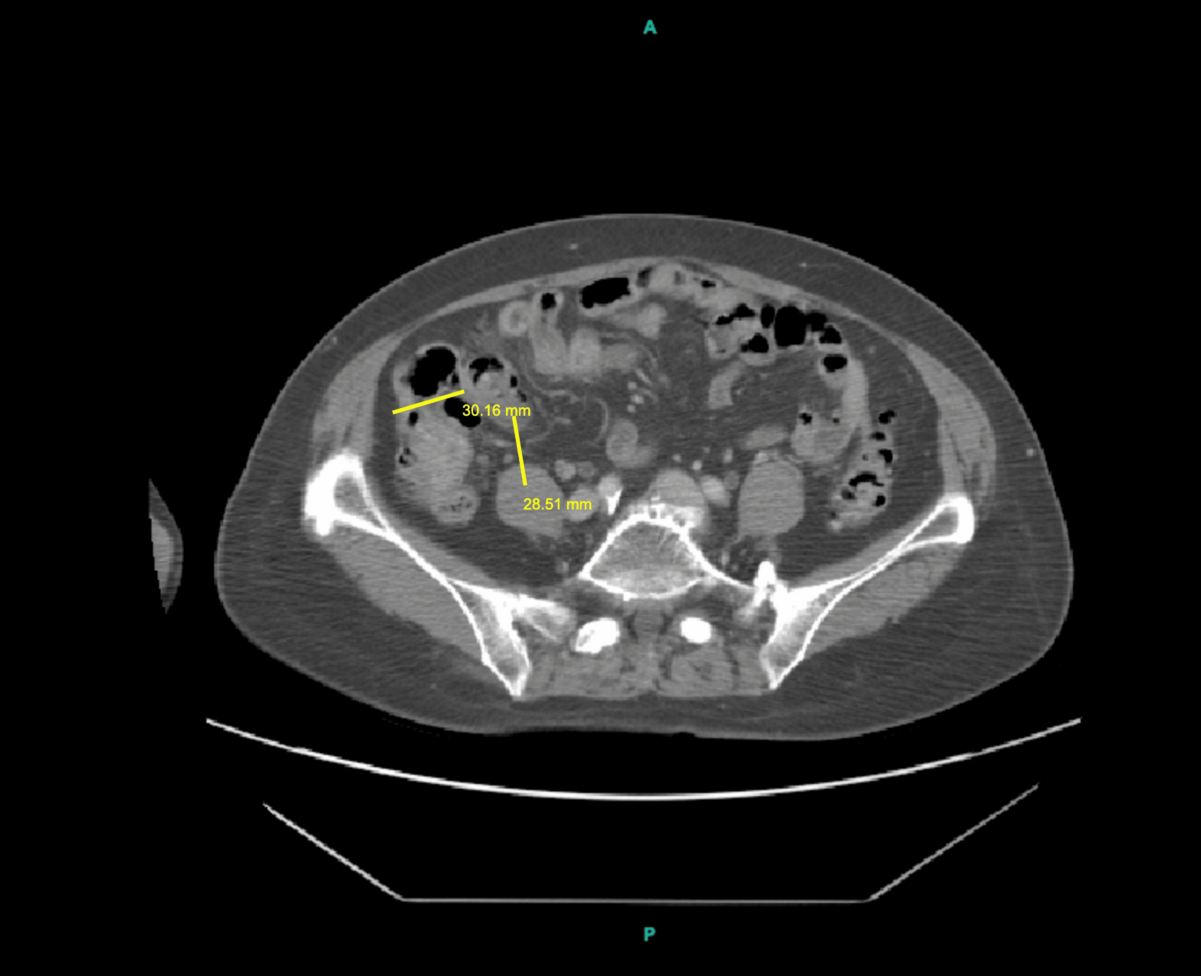
An axial CT scan of the abdomen demonstrating the ascending colon with a suspected polypoidal lesion. The yellow lines correspond to the dimensions of the polyp: the 30.16 mm line (anterior to the polyp) represents the transverse (left-right) axis, while the 28.51 mm line (medial to the polyp) represents the AP axis. CT: computed tomography; AP: anteroposterior

A follow-up computed tomography (CT) scan of the abdomen and pelvis, performed one week after the colonoscopy, revealed a non-metastatic mass in the ascending colon (Figure 1). Additionally, gallstones were identified, correlating with the patient's reported abdominal discomfort (Figure 2). Given the size of the sessile polyp and the associated findings, a surgical intervention was deemed necessary.

After discussing the risks, benefits, and alternatives with the patient and her daughter, consent was obtained to proceed with a single-incision laparoscopic right hemicolectomy in combination with a single-incision laparoscopic cholecystectomy at Advanced Surgical Associates in California. This dual approach was recommended to address both the colonic mass and symptomatic cholelithiasis in a single surgical session, thereby minimizing the need for subsequent surgeries and reducing the risk of postoperative adhesions.

After thorough explanations of potential complications, including anastomotic leak, bleeding, infection, and injury to adjacent organs, informed consent was obtained. The patient was scheduled for follow-up in the surgical clinic two weeks post-operation to assess recovery and monitor for any complications.

Surgical procedure

**Figure 3:**
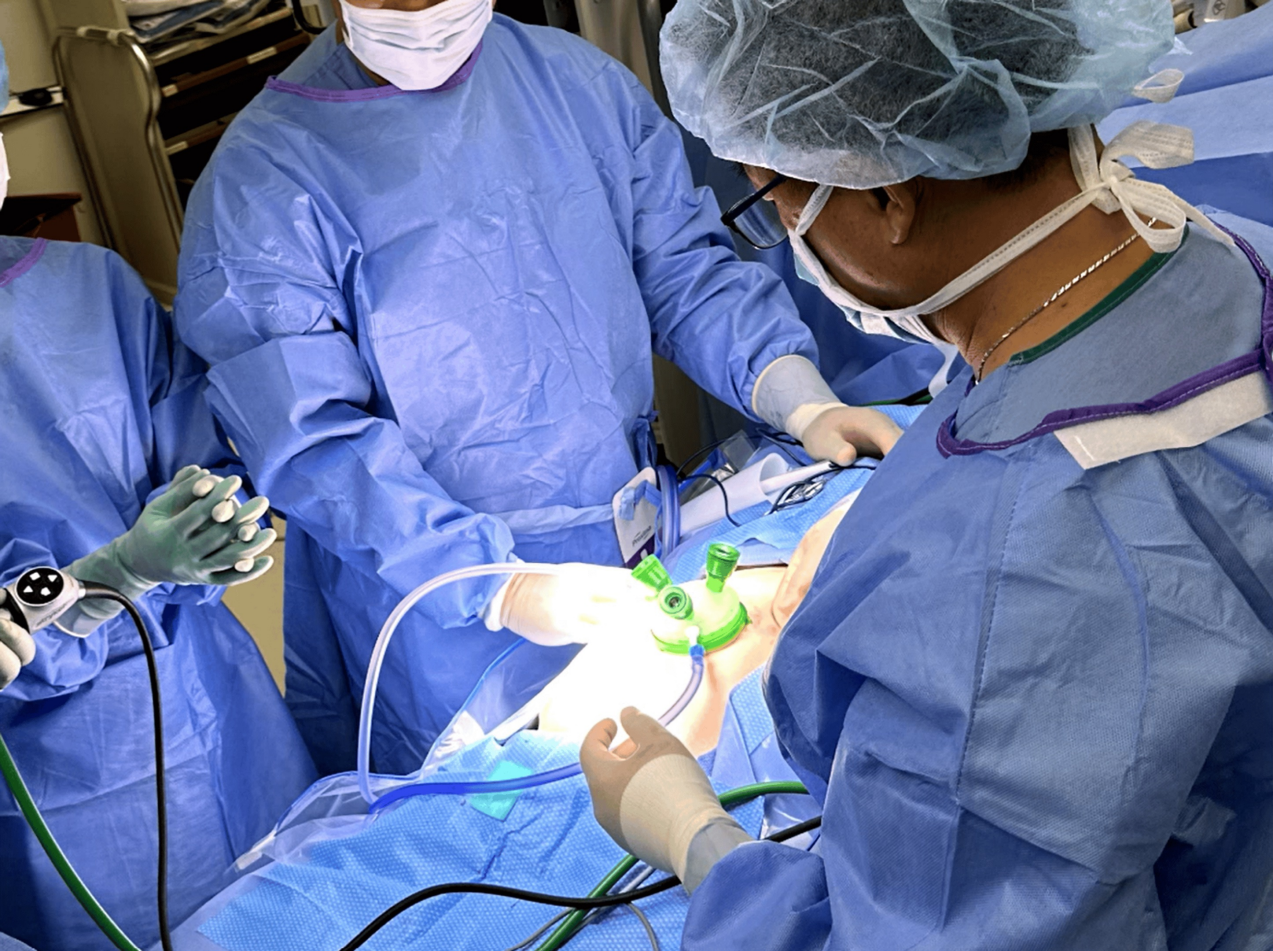
Advanced access platform application following a single incision at the umbilicus

**Figure 4:**
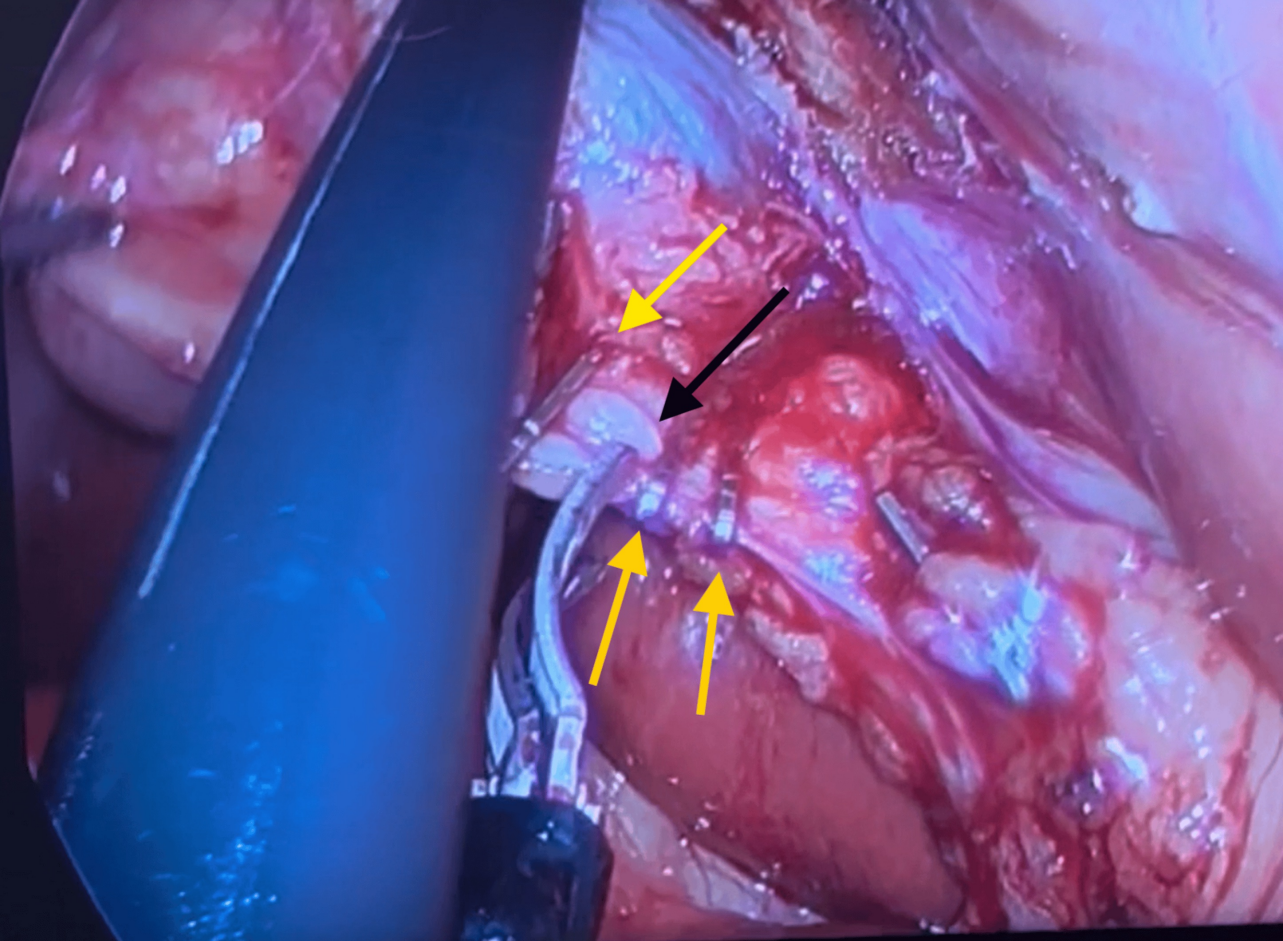
Cholecystectomy procedure: yellow arrows demarking staples for ligation and the black arrow demarking the division of the cystic duct between the second and third staples

**Figure 5:**
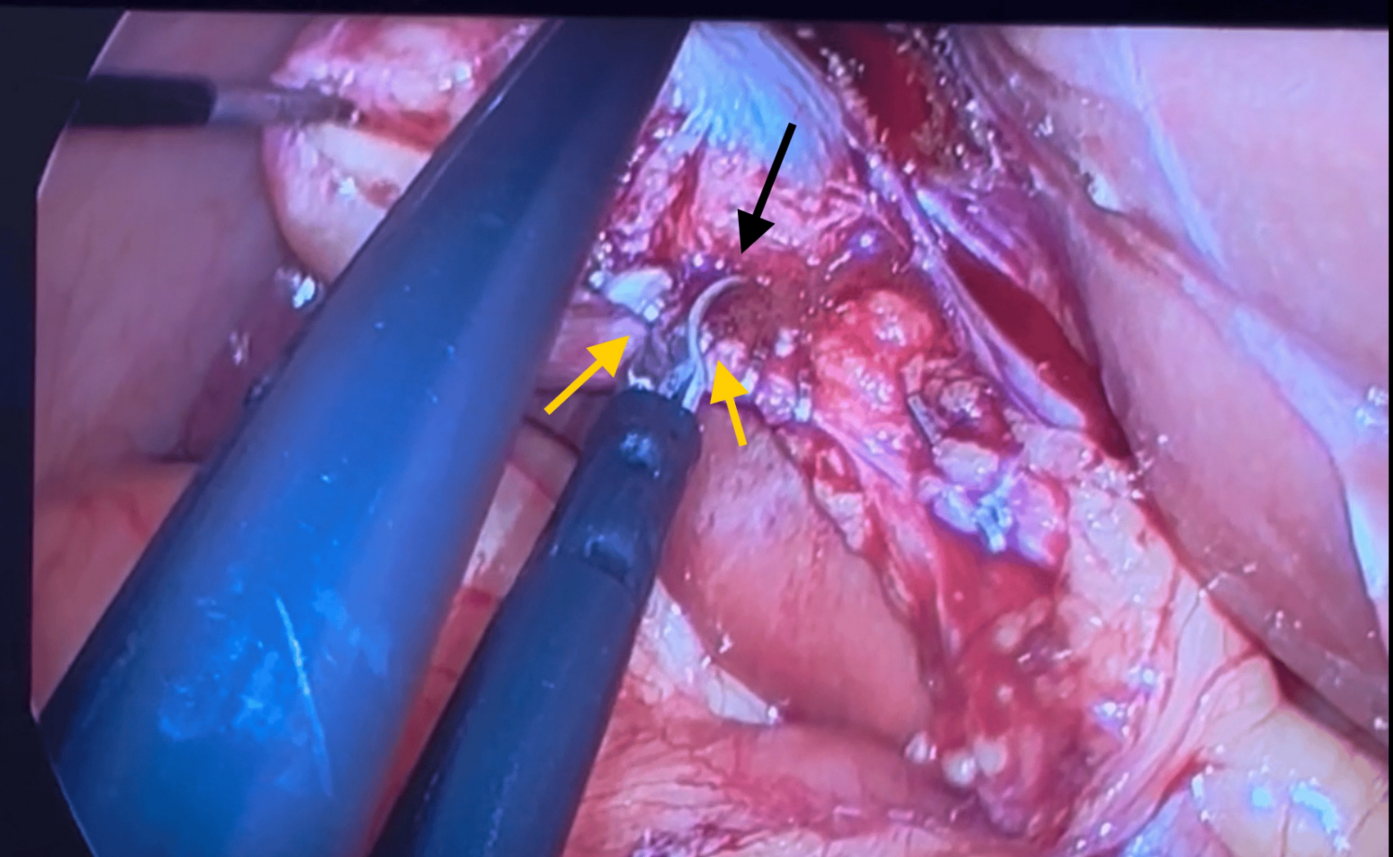
Cholecystectomy procedure: yellow arrows demarking staples for ligation and the black arrow demarking the division of the cystic artery

The patient was placed in the supine position and under general anesthesia with endotracheal tubing and urethral catheterization. The abdomen was painted with Betadine, and sterile draping was applied. A 3-cm single incision was made at the umbilicus and carried down through the fascia until the abdomen was entered. A GelPOINT Mini advanced access platform (Applied Medical Resources Corporation, Rancho Santa Margarita, CA, USA) was inserted, and a laparoscope was pushed into the trocar through the advanced access platform (Figure 3). The cholecystectomy was performed first. The gallbladder was grasped and retracted anteriorly using an Endo Babcock (Medtronic, Minneapolis, MN, USA). Calot's triangle was identified, and the cystic duct was bluntly dissected, isolated, doubly ligated with 5 mm clips, and divided (Figure 4). A cystic artery was also found to be doubly ligated with 5 mm clips and divided (Figure 5). An advanced access platform alligator was used to further retract the fundus of the gallbladder. The Hartmann pouch was retracted outwardly. The gallbladder was then meticulously dissected off the liver bed, and hemostasis was applied with the use of electrocautery. A review of the liver bed and surgical site revealed no active oozing or bleeding, and the gallbladder was then removed from the abdomen via the single-incision advanced access platform site.

**Figure 6:**
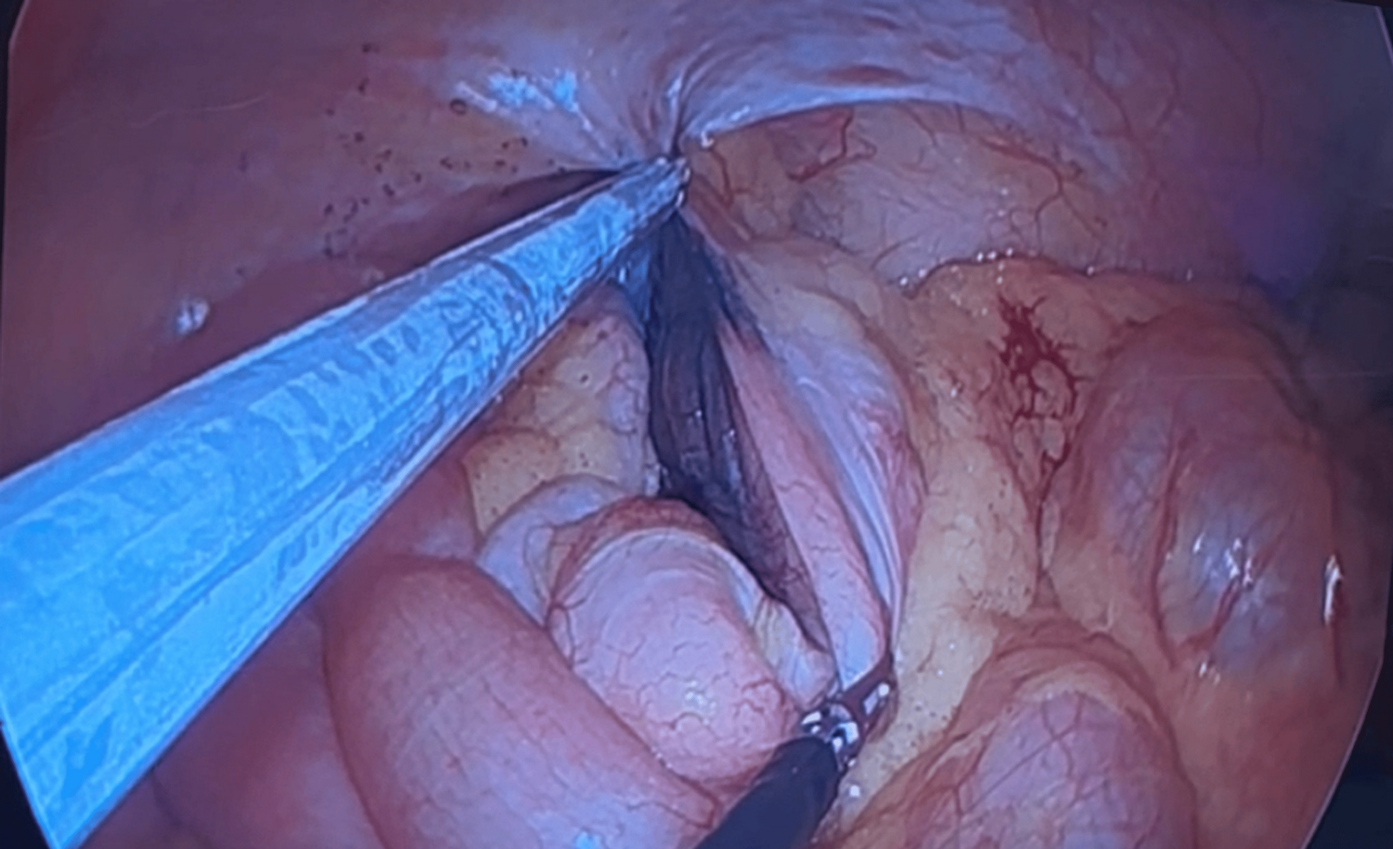
Hemicolectomy procedure: visualization of the India ink tattoo demarking the site of the ascending colon polyp found on colonoscopy

**Figure 7:**
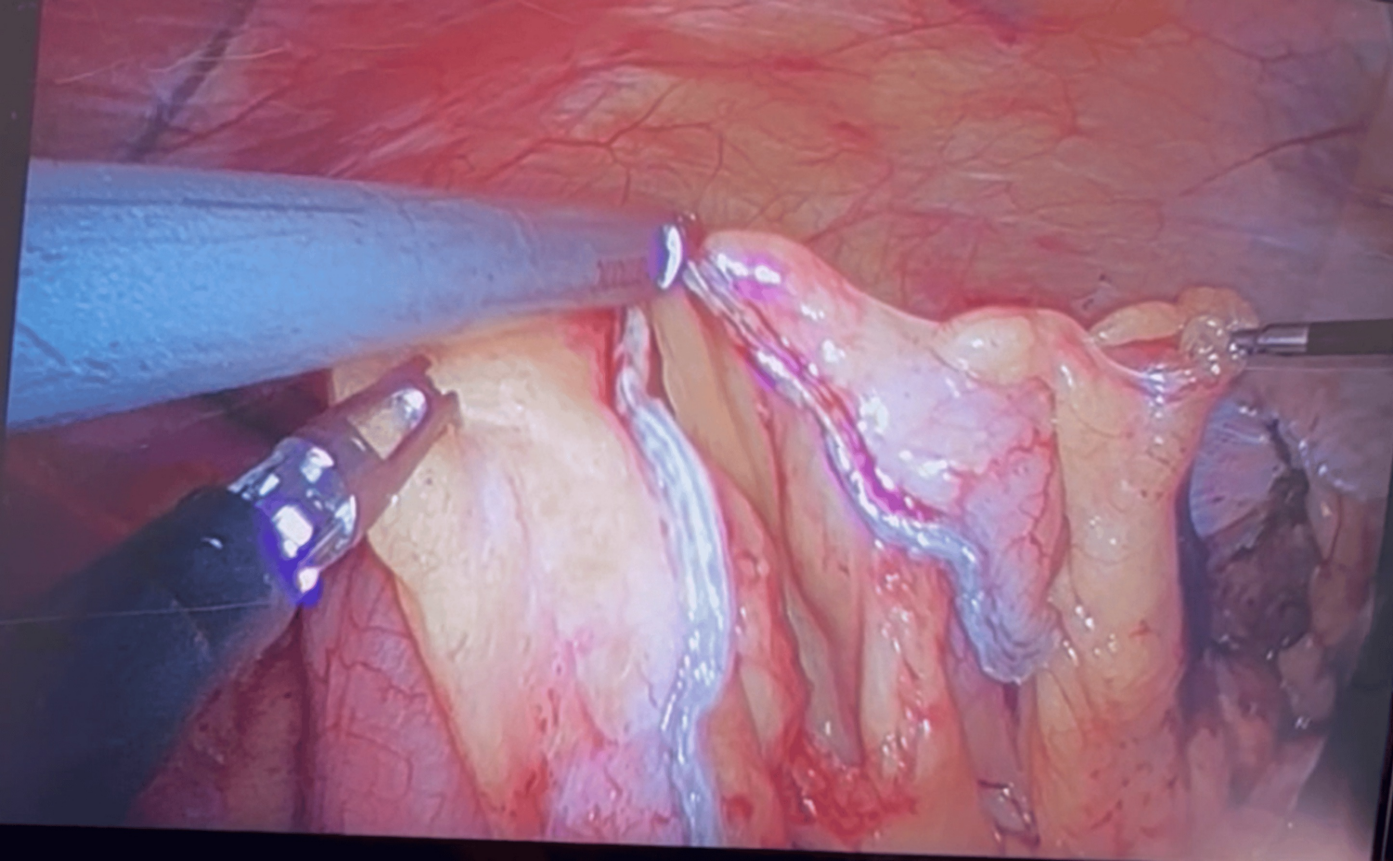
Hemicolectomy procedure: division of the terminal ileum via Endo-GIA

**Figure 8:**
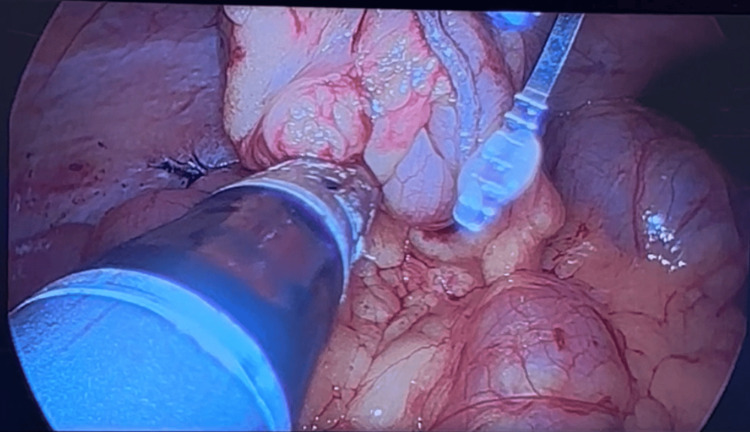
Hemicolectomy procedure: division of the right mesocolon and right colic vessel via Endo-GIA

**Figure 9:**
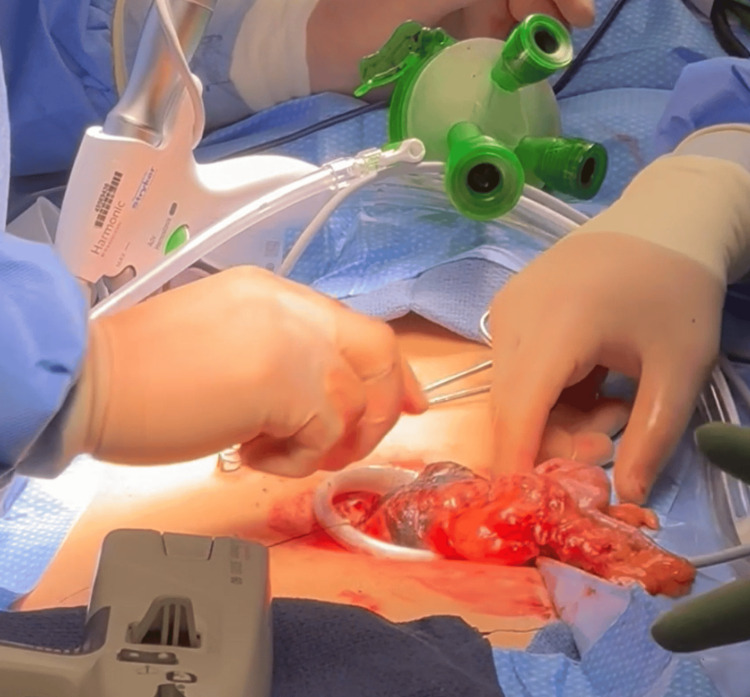
Hemicolectomy procedure: removal of the right colon through the single incision site

**Figure 10:**
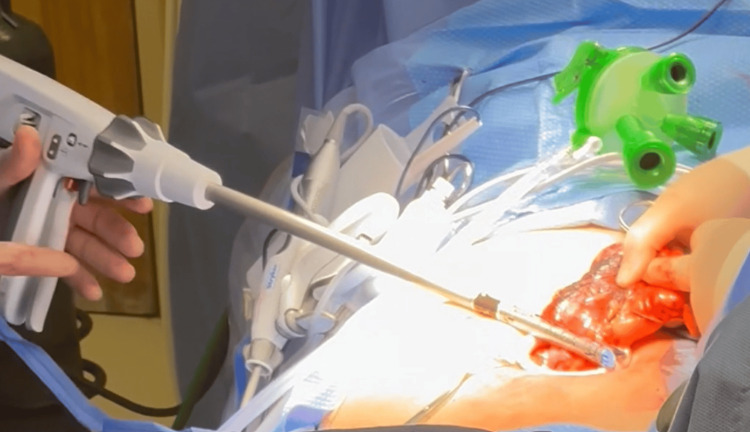
Hemicolectomy procedure: division of the right colon distal to the India ink tattoo with Power GIA stapler

**Figure 11:**
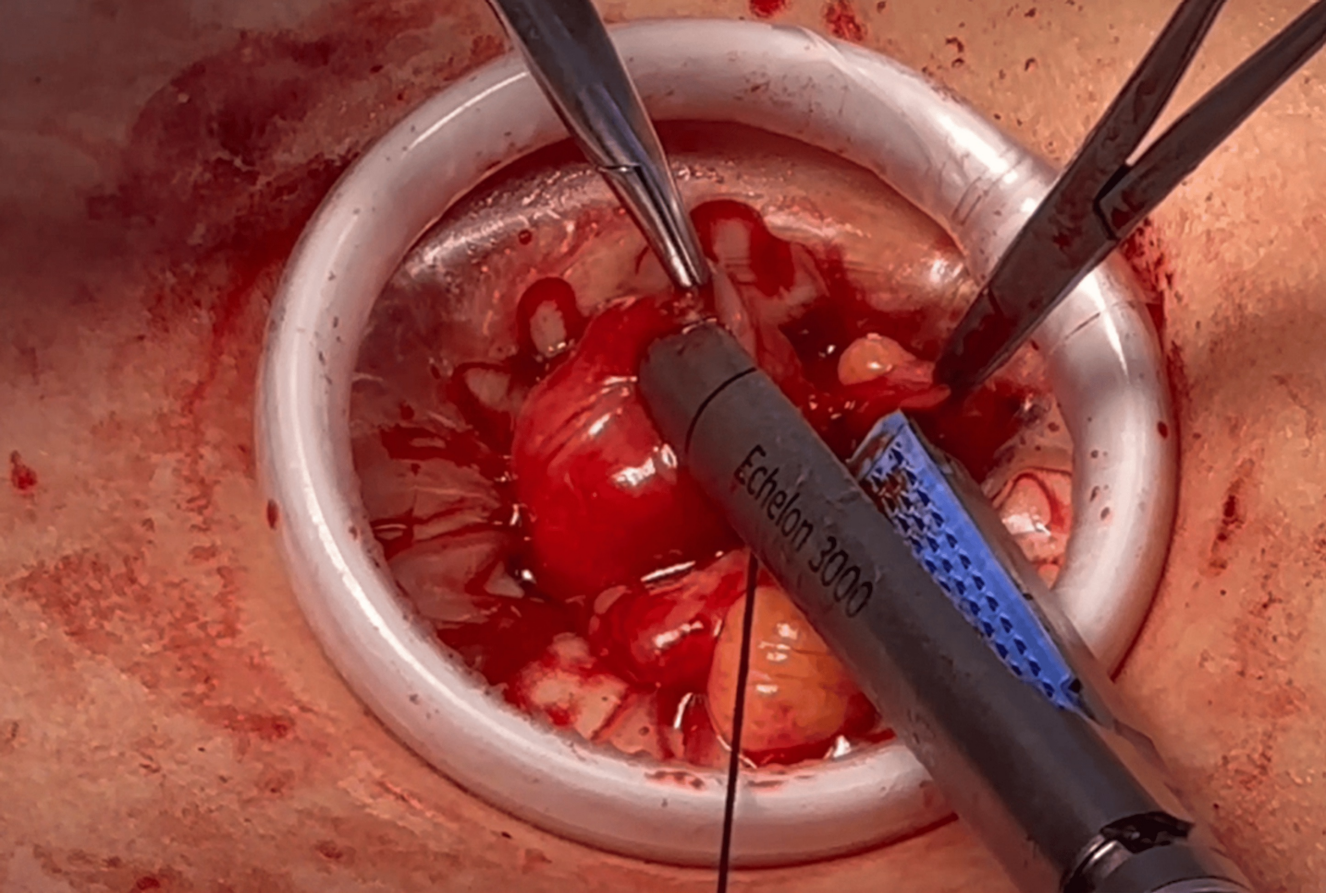
Hemicolectomy procedure: side-to-side anastomosis created with Endo-GIA

**Figure 12:**
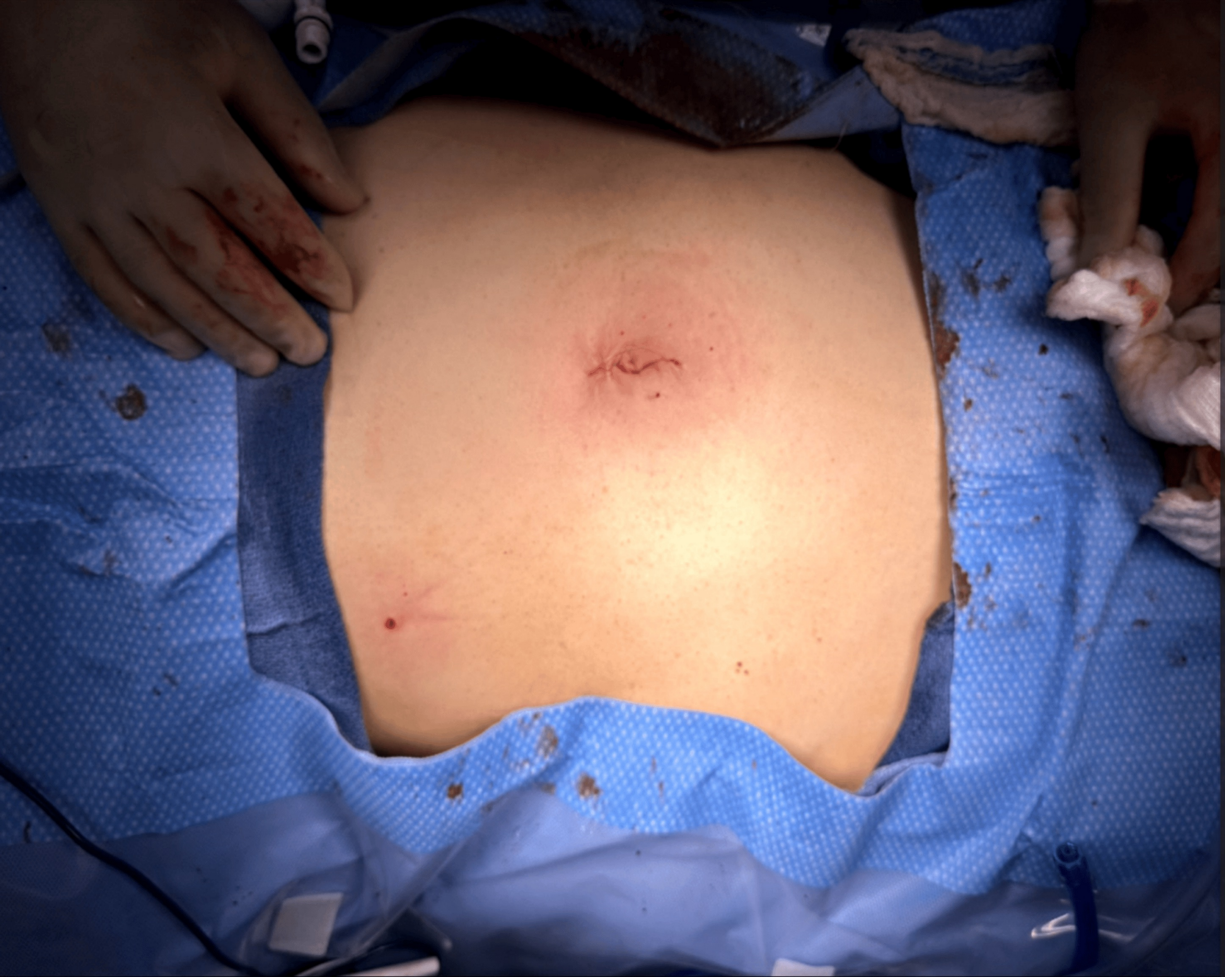
Closure of the single incision

Following the successful completion of the cholecystectomy, the single advanced access platform was reestablished, and the abdomen was re-insufflated in preparation for the right hemicolectomy. Over the ascending colon proximal to the cecum, India ink tattoo was visualized distally and proximally (Figure 6). Mobilization of the cecum away from the right lateral wall was established by incising the line of Toldt. Additionally, the hepatocolic ligaments were bluntly and sharply divided distal to the hepatic flexure to mobilize the right colon away from the right gutters. The terminal ileum was divided with an Endo-GIA (Medtronic, Minneapolis, MN, USA) (Figure 7). The mesoappendiceal artery was then divided using a GIA stapler, and the right mesocolon, along with the right colic vessel, was also divided with the use of a vascular GIA down to the root of the mesentery (Figure 8). The specimen was then removed from the abdomen (Figure 9). The specimen was divided distal to the India ink tattoo via the Endo-GIA and was sent for pathological review (Figure 10). The terminal ileum was also brought out for extracorporeal side-to-side anastomosis with a GIA stapler (Figure 11). The stump was oversewn with an inner layer of 3-0 Chromic and an outer layer of 2-0 Silk in an interrupted suture. The anastomosis was complete and pushed back into the abdominal cavity. Copious amounts of irrigation were supplied, revealing no sign of bleeding or oozing. The liver and the rest of the abdomen appeared normal. The advanced access platform was removed, and the abdomen was desufflated. The single incision at the umbilicus was closed with a running 3-0 Chromic, and the skin was closed with 3-0 Vicryl (Figure 12). The patient tolerated the procedure without any complications and was transferred to the recovery room. All the authors of this report actively participated in or observed the surgical procedure and contributed to the postoperative follow-up of the patient.

Results

The procedure was completed in 90 minutes without significant complications and minimal blood loss. Additional laparoscopic ports were not required. The pathologic report showed a gallbladder with multiple gallstones measuring up to 0.5 cm, consistent with chronic cholecystitis and cholelithiasis. The report also showed a 1.8 cm sessile polyp at the ascending colon with negative margins; sections of the polyp show a tubulovillous adenoma completely excised, with focal early cribriform architecture seen without definite high-grade cytologic features. A total of 15 lymph nodes were excised without evidence of metastatic carcinoma.

**Figure 13:**
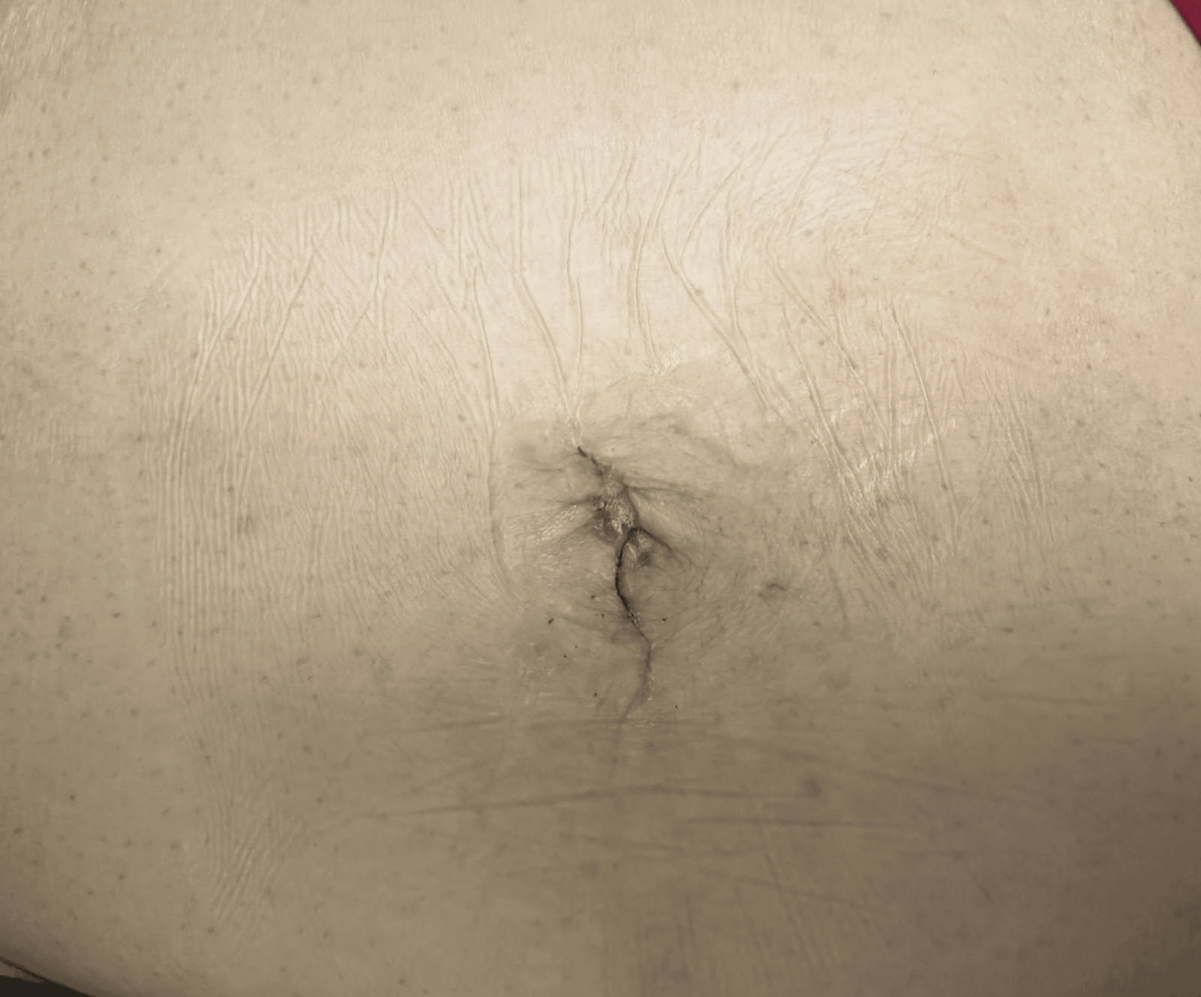
Postoperative evaluation of the single incision site

The patient was able to get out of bed and ambulate postoperatively that afternoon, pass gas on postoperative day three, start a clear liquid diet, and was cleared for discharge on postoperative day four without any complications. On postoperative day seven, the patient resumed a regular diet and reported resuming normal daily activities without any signs of complications such as surgical site infection by postoperative day 15. The patient was ecstatic by the cosmetic results (Figure 13) and rapid recovery progression.

## Discussion

SILS for right hemicolectomy and SILS for cholecystectomy have been well-described in the literature; however, we are the first group to report a combined single-incision cholecystectomy and right hemicolectomy. By simultaneously performing both procedures through a single incision, we spare the patient from a second abdominal surgery and its associated risks. Although a prior history of abdominal surgeries is no longer considered a contraindication for laparoscopic procedures, it is still recognized as a significant risk factor for increasing postoperative complications, such as inadvertent enterotomy, postoperative ileus, and higher conversion rates [6]. For example, the development of postoperative adhesions can pose challenges during repeated abdominal surgeries, including increased operative time, site access difficulties, higher rates of hospital readmission, and the risk of intestinal obstruction [7,8]. These complications can be mitigated in patients requiring multiple procedures by utilizing a combined SILS approach.

Typically, a single-incision cholecystectomy lasts 42-45 minutes versus 59-62 minutes for a standard laparoscopic cholecystectomy (SLC), while a single-incision right hemicolectomy performed in Chow et al. took 175 minutes versus 197 minutes for conventional laparoscopic surgery [2,9,10]. In this report, the combined SILS procedure was completed in 90 minutes, demonstrating a significant reduction in operative time. Although it is recognized that operative time may be influenced by the surgeon's extensive surgical skills and experience with SILS, this does not negate the potential to reduce overall operative time.

Many studies have demonstrated the potential of SILS to decrease postoperative pain, blood loss, operating time, and hospital stay, with low complication rates and better cosmetic outcomes [9,11,12]. In contrast, a meta-analysis by Trastulli et al. and a retrospective study by Culp et al. reported significantly higher procedural failure rates, increased blood loss, and longer operating times compared to SLC, respectively [13,14]. These discrepancies can be attributed to the surgeon's skill level and experience with SILS. Due to the "sword fighting" nature of SILS, including the loss of triangulation, restrictive movements, lack of additional retraction, and depth perception changes from camera positioning, surgeons must overcome a steep learning curve (LC) before realizing the benefits. One study reported that 10-15 single-incision cholecystectomy cases were needed before achieving comparable operating times to SLC, while another study suggested that 25 cases were required [15,16].

In a multicenter, retrospective study conducted by the Nagasaki Colorectal Oncology Group (NCOG), the use of an organ retractor was shown to potentially decrease the LC in patients with right-sided colon cancer. The study reported a statistically significant decrease in operative time to 117 minutes and blood loss in SILS compared to the conventional laparoscopic approach, even in low-volume centers that perform fewer than 200 colorectal cancer (CRC) surgeries annually [10]. Additionally, the literature suggests that intraoperative restrictions, particularly with anterior resection, can be overcome through the use of SILS plus a one-assist port (SILS+1), thereby further lowering the LC [17,18].

With each passing year, more SILSs are being performed, and various interventions are being implemented to reduce the LC associated with the technique. Nonetheless, further research is necessary to thoroughly quantify and justify the practicality of SILS in comparison to conventional laparoscopic techniques.

## Conclusions

This case report demonstrates the successful combination of SILS for right hemicolectomy and cholecystectomy in a single procedure. For our patient, this approach effectively avoided the need for multiple surgeries, minimized surgical trauma, maintained a reasonable operating time, and led to a smooth recovery, all while achieving excellent cosmetic results. Although these outcomes were favorable in this case, further research and clinical trials are necessary to determine the broader applicability of this technique, especially in more complex cases.
